# Utility of Computational Methods to Identify the Apoptosis Machinery in Unicellular Eukaryotes

**DOI:** 10.4137/bbi.s430

**Published:** 2008-03-12

**Authors:** Pierre Marcel Durand, Theresa Louise Coetzer

**Affiliations:** Department of Molecular Medicine and Haematology, University of the Witwatersrand and National Health Laboratory Service, Johannesburg, South Africa

**Keywords:** bioinformatics, programmed cell death, apoptosis, protozoa

## Abstract

Apoptosis is the phenotypic result of an active, regulated process of self-destruction. Following various cellular insults, apoptosis has been demonstrated in numerous unicellular eukaryotes, but very little is known about the genes and proteins that initiate and execute this process in this group of organisms. A bioinformatic approach presents an array of powerful methods to direct investigators in the identification of the apoptosis machinery in protozoans. In this review, we discuss some of the available computational methods and illustrate how they may be applied using the identification of a *Plasmodium falciparum* metacaspase gene as an example.

Bioinformatics has become an increasingly useful, and for genomic and proteomic analysis indispensable, tool in molecular biology research. Due to the nature of genomic and protein sequence information, bioinformatics forms part of almost any molecular biology research program. In this paper, we discuss the application of computational methods to the study of programmed cell death (PCD) in protozoan biology. An exhaustive list and discussion of all available computational methods is not feasible and we have chosen to focus on the most useful and frequently used methods. But first, a brief overview of PCD and the evidence for this process in protozoa are necessary.

## Programmed Cell Death: An Overview

PCD refers to the inherent capacity of each cell to follow an active and regulated process of self-destruction and is considered the “default” state of a cell (a list of key terms used in this review may be found in Table 1). For a detailed review of PCD from an evolutionary perspective see Ameisen (2002). Depending on signals from other cells or the environment and the integrity of its internal state, a cell either follows a path of self-destruction or this pathway is actively repressed and the cell continues with its functions. The process of cell death may be reversible early on, but at some stage the cell continues irreversibly towards self-destruction. Once the cell reaches a point where the features of cell death become apparent, it is said to be undergoing apoptosis. Although the terms “programmed cell death” and “apoptosis” are sometimes used synonymously, this is incorrect since apoptosis, although the most typical phenotype of PCD, is not the only one. Other PCD phenotypes, also called “morphotypes”, include autophagy, oncosis, pyroptosis and paraptosis all of which differ in their biochemical or morphological properties (Fink and Cooksen, 2005; Sperandio et al. 2004). Autophagy describes cell death characterized by the formation of intracellular vesicles known as autophagosomes, consisting of sequestrated cell debris. The sequestration process is regulated by GTPases, phosphatidylinositol kinases and ubiquitin-like conjugation systems none of which play a part in apoptosis. Oncosis is a regulated response to severe DNA injury leading to cellular energy depletion, organelle swelling and cell membrane disruption. Pyroptosis denotes a pro-inflammatory cell death pathway different from the anti-inflammatory pathways of apoptosis and is induced by intracellular bacteria in phagocytic cells. Paraptosis is morphologically similar to apoptosis but again, employs a different biochemical pathway. Some of these alternate forms of PCD, such as paraptosis, have been described in unicellular eukaryotes, for example *Amphidium carterae* (Franklin and Berges, 2004). However, the focus of this review is on the molecular machinery involved in apoptosis.

**Figure 1:**
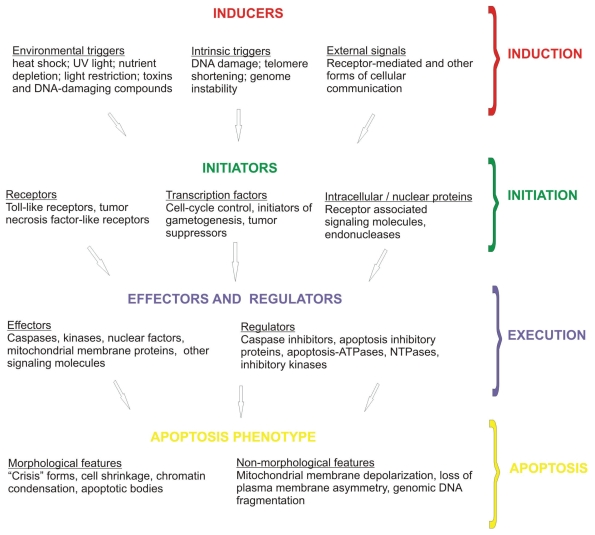
Four representative stages of Programmed Cell Death.

To investigate the apoptosis machinery in protozoans, we propose that PCD be viewed in four stages to make it more amenable to study by computational methods: induction, initiation, execution and apoptosis (Fig. 1). Stellar (1995) and Jabs (1999) provide useful overviews of the mechanisms of apoptosis. The four stages overlap and are somewhat artificial but the distinction will assist a bioinformatic approach. PCD requires one or more stimuli (inducers) for the process to begin by induction, the nature of which depends upon the organism but the end point is an evolutionarily determined recognition by the organism that continued life in the present form is undesirable. In protozoa inducers are frequently environmental signals like light-deprivation (Segovia et al. 2003), heat-shock (Lee et al. 2002), or nutrient depletion (Cornillon et al. 1994). Induction may also simply be the absence of a signal that represses the default program to apoptose. An organism’s response to induction is initiation of PCD. Initiators are the genes and proteins responsible for detecting induction and lead to a state of quiescence (a reversible inactive state) or directly on a path of self-destruction. Initiators may be receptors, transcription factors, enzymes or other proteins involved in protein-protein interactions (Aravind et al. 1999).

Once the decision to self-destruct has been made, the third stage in PCD, execution, is implemented. This involves a complex cascade and network of interacting proteins which is necessary for amplification and regulation of the process (for a review see Koonin and Aravind, 2002). Multiple protein families are involved in the execution stage but they may be broadly allocated into two groups: effectors and regulators. Effectors include caspases, kinases, phosphatases, Apoptosis-ATPases (Ap-ATPases) and other NTPases. The caspases belong to a superfamily of proteases and are the major effectors of PCD. They are all cysteine proteases and cleave specific aspartate-containing sites in other proteins of the apoptosis cascade. Kinases and phosphatases activate or inactivate other protein targets via phosphorylation and dephosphorylation. Caspases, kinases and various other proteins in PCD pathways, such as receptors and their associated signaling molecules, contain evolutionarily conserved domains known as “adaptors” that are essential for protein-protein interactions. These adaptor domains are themselves of various classes such as the death domain (DD), death-effector domain (DED) and caspase activation and recruitment domain (CARD). Effectors may contain one or more adaptor domains in their structure. Conserved domains have also been found in some of the regulators of the execution stage. The family members known as inhibitor of apoptosis proteins (IAPs) directly inhibit caspases and also contain conserved domains such as the CARD domain. Functionally inactive caspase homologs have been discovered which act as dominant negative molecules thereby downregulating the caspase cascade. Regulators may also be found embedded in the outer mitochondrial membrane where they exhibit anti-apoptotic (e.g. BCL-2) or pro-apoptotic (e.g. BAX) activity and interact with Ap-ATPases. Ap-ATPases belong to a phylogenetically-diverse superfamily of proteins that are uniquely defined by five conserved motifs and have various regulatory functions in PCD. Finally, there are other NTPases, serine/threonine kinases and many other miscellaneous regulators some of which contain conserved domains amenable to bioinformatic analysis.

The culmination of PCD is the fourth and final stage of self-destruction, the most typical phenotype of which is apoptosis. The microscopic features of apoptosis are cell shrinkage, membrane blebbing, chromatin condensation and a pyknotic nucleus, and formation of apoptotic bodies. These features may not be specific to apoptosis and more objective methods exist such as measurements of mitochondrial membrane depolarization, loss of plasma membrane asymmetry, and genomic DNA fragmentation.

## Unicellular Eukaryotes and Self-Destruction

The study of PCD revealed the central role of this process in development and tissue homeostasis. PCD was considered a hallmark of multicellularity, but has subsequently been demonstrated in phylogenetically diverse organisms, including protozoa, suggesting that its origins are evolutionarily ancient and predate multicellularity (Segovia et al. 2003). With the notable exception of metacaspase proteins, however, *in silico* studies of protozoan genomes have failed to detect the key components of apoptosis such as Bcl-2, BAX, apoptosis-inducing factor and other effectors and regulators. The reasons for this are not known but there are a few possibilities. Due to the evolutionarily diverse nature of the protozoans investigated, the sensitivity and specificity of the homology searches used may be inadequate. The unusual features of some of the genomes such as the nucleotide bias in *P. falciparum*, may further impact negatively on the bioinformatic methods used. It is also possible that homologs of some of the proteins involved in PCD in animals, plants and fungi are simply not present in protozoans, which may be employing unknown pathways. In the case of parasitic protozoa, this would be important for the development of novel drug targets.

The evolutionary reasons for PCD in unicellular eukaryotes are unknown and raise intriguing questions regarding their survival strategies. It is likely that the selective forces for PCD in protozoa differ between organisms. As an example, PCD has been demonstrated in *Plasmodium* ookinetes in its mosquito host (Al-Olayan et al. 2002), which may be important to limit the severity of the infection, thereby improving the parasite’s chances of transmission (Deponte and Becker, 2004). Identification and investigation of the apoptosis machinery in protozoa are likely to provide more insight into this phenomenon. For further consideration of this topic, the reader is referred to Ameisen (2002) and Koonin and Aravind (2002).

The evidence for PCD in unicellular eukaryotes (Kingdom: Protista or Protozoa) has largely come in the form of laboratory identification of the inducers of PCD and demonstrations of the apoptosis phenotype. Apoptosis has been demonstrated in a wide range of protozoa: the kinetoplastid parasites *Trypanosoma* (Ameisen et al. 1995) and *Leishmania* (Moreira et al. 1996), which are among the most ancient eukaryotes; the slime mold *Dictyostelium discoideum* (Cornillon et al. 1994), which has a more recent origin; the free living ciliate *Tetrahymena thermophila* (Christensen et al. 1995); *Peridinium gatunense* (Vardi et al. 1999) and other dinoflagellates (Dunn et al. 2004); two apicoplast parasites of the genus *Plasmodium* (Al-Olayan et al. 2002; Meslin et al. 2007); the parasite *Titrichomonas foetus* (Mariante et al. 2006); the chlorophyte *Dunaliella tertiolecta* (Segovia et al. 2003); the amoeba *Entamoeba histolytica* (Mendoza et al. 2003); and the intestinal parasite *Blastocystis hominis* (Nasiruden et al. 2001). While progress has been made in identifying the inducers of PCD and the features of apoptosis, almost nothing is known about the genetic and protein machinery that comprise the initiators and executors of PCD in protozoa. In fact, a survey of the literature suggests that more is known about homologs of the apoptosis machinery in plants, fungi and bacteria than protists.

## A Bioinformatic Approach to Investigating Apoptosis in Protozoans

A bioinformatic approach is well suited for directing investigations into the second (initiation) and third (execution) stages of PCD. The aim of this approach is to identify the genes and proteins involved in PCD based upon their evolutionary relatedness to known apoptosis machinery molecules. A summary of the key components of the apoptosis machinery in multicellular animals is listed in Table 2 and highlights the conserved regions that may be used to identify homologous components.

### Domains versus whole proteins

It is usually more appropriate to apply bioinformatic methods to domains rather than whole genes or proteins for a number of reasons. Recombination events may lead to homologous sequences not being detected due to the insertion of unrelated sequences; or to unrelated sequences being erroneously identified as homologs when small stretches of evolutionary related sequences are inserted. These disruptive effects that result from recombination events are diminished when domains rather than complete sequences are used. Domains may also be considered the evolutionary unit (Gabaldon, 2005) since they evolve at different rates within the same protein based on their function. The use of domains will improve both the sensitivity and specificity of the methods employed. Furthermore in the case of identifying the initiators and executors involved in PCD in protozoans, there is considerable overlap among the conserved domains in many of the apoptosis proteins, for example the DD, DED and CARD domains are found in receptors, receptor associated proteins and signaling molecules, kinases, caspases and regulatory proteins (Aravind et al. 1999). It makes more sense therefore to make use of the conserved nature of these domains in a search for related sequences.

### Methodology

**Figure 2:**
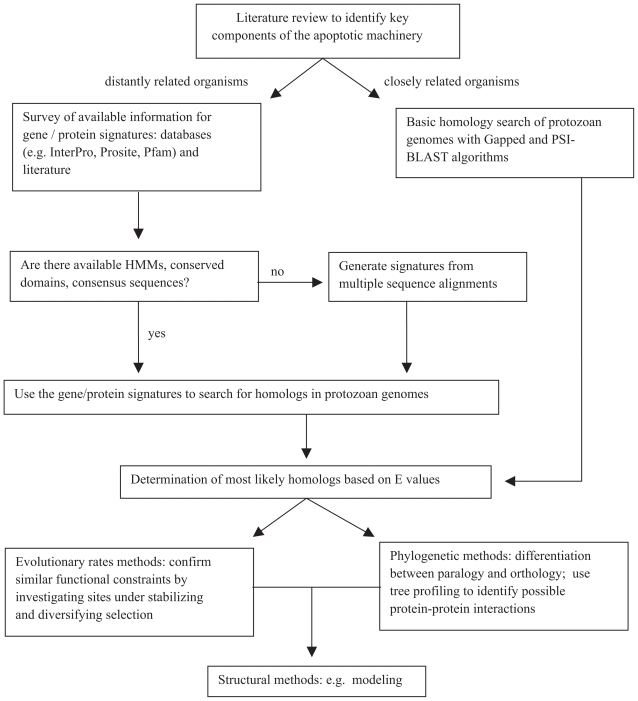
A computational approach to the identification and characterization of apoptosis homologs in unicellular organisms.

**Figure 3:**
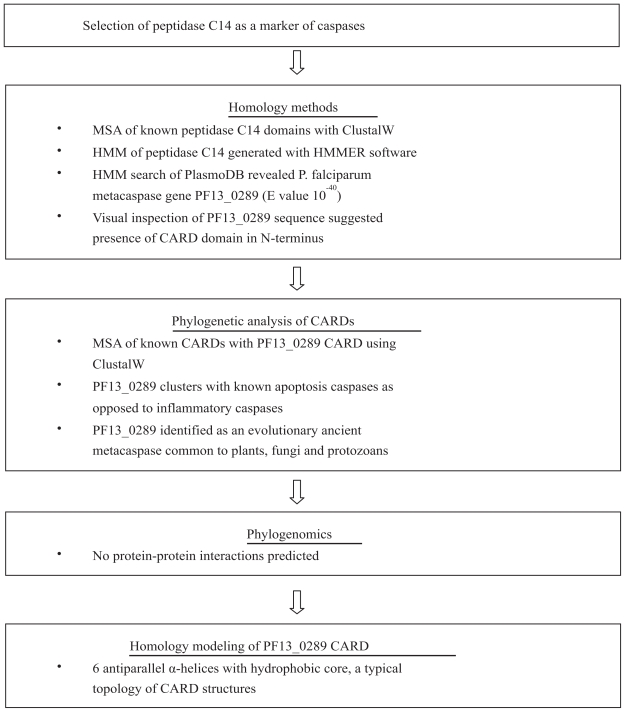
The bioinformatic steps taken to identify and investigate a *P. falciparum* metacaspase gene (PF13_0289). The mirror tree, gene fusion and gene neighborhood methods for determining protein-protein interactions, and sequence evolution analysis were not performed. **Abbreviations:** CARD: caspase activation and recruitment domain; HMM: hidden Markov model; MSA: multiple sequence alignment.

An array of bioinformatic techniques is available. We have selected several methods and suggest how they may be implemented to give the investigator the greatest chance of success. The following methods will be discussed i) homology-based methods including the use of hidden Markov models; ii) phylogenetics; iii) phylogenomics and whole genome methods; iv) sequence evolution; and v) structural methods. Table 3 provides a list of computational methods that may be used to identify and investigate homologous genes and proteins and Figure 2 is a flow diagram indicating how they may be implemented. Some of these methods have been used to uncover a *Plasmodium* metacaspase gene (Fig. 3) and where appropriate, this is presented as an example of how bioinformatics may be applied to identify and investigate the apoptosis machinery in protozoans. A list of useful websites and databases may be found in Table 4.

## Homology Methods

### Sequence alignments

The bioinformatic identification of orthologs frequently depends upon sequence homology since “similar sequence” usually implies “similar function”. As mentioned above, the evolutionarily conserved regions, such as the DD, DED or CARD domains in caspases, the zinc finger domains in IAPs, the five conserved motifs in Ap-ATPases, and the conserved regions in transcription factors and receptors are natural starting points for ortholog identification. For straightforward searches where a high degree of similarity is expected, the FASTA and BLAST programs are useful. This may be applied to some unicellular eukaryotes once orthologs have been identified in more closely related organisms, but currently, the evolutionary distances are too great and a more powerful method is required. The Gapped and Position-Specific Iterated BLAST (PSI-BLAST) programs are profile-based methods that more than double the sensitivity of homology searches (Park et al. 1998) by taking into account the possibilities of insertions/deletions in related sequences which generate gaps in an alignment (Gapped BLAST), or incorporating information embedded in a multiple sequence alignment into a position-dependant weight matrix (PSI-BLAST) (Altschul SF et al. 1997). Gapped BLAST and PSI-BLAST allow the user to lower the threshold (T) value to improve sensitivity. Caution needs to be exercised however as PSI-BLAST amplifies any errors introduced in the first iteration. It is advisable therefore to corroborate findings after the first iteration by close inspection of alignments before continuing. Care must also be taken when searching genomes or proteomes with biased compositions (see “Conventional methods and unconventional genomes” below). Examples of biases include repetitive elements or a skewed nucleotide composition in genes, and low complexity regions in proteins. If a biased region forms part of a sequence profile, unrelated sequences with a similar bias may be introduced during subsequent iterations rendering the analysis meaningless. PSI-BLAST uses a default program to filter out biased regions but this only detects a gross bias. Manual inspection will usually identify results with biases that are not filtered out.

A multiple sequence alignment (MSA) is usually necessary before any of the methods discussed below can be implemented and is therefore a critical step. The aim of any alignment procedure is to maximize the homology. Various automated procedures are available which differ in their accuracy when aligning large data sets, complex sequences, highly divergent taxa, dealing with indels (insertions and deletions), and in computational efficiency. Some examples of the algorithms to perform MSAs are CLUSTAL, Muscle, T-Coffee, PileUp and MAFFT (for a review of MSA methods see Morrison, 2006 and Martin et al. 2007). Depending on the data, not all algorithms are guaranteed to give optimal results. In addition, changing the alignment parameters (such as the Gap penalty) or specifying certain assumptions (such as the presence of low complexity sequences interrupting a conserved domain) are likely to give different results.

### Hidden Markov models

MSAs of orthologs may be used to generate patterns of conservation or signature profiles of a specific gene or protein. Hidden Markov models (HMMs) are examples of such molecular signatures and are very informative for identifying distantly related sequences (profile hidden Markov models are reviewed in Eddy, 1998). This will be a useful method for identifying the apoptosis machinery in unicellular eukaryotes where very little is known about the genes and proteins involved in these organisms. A HMM describes a probability distribution over a potentially infinite number of states. It makes use the Markov property which assumes that a present state is conditionally independent of any prior states, that is, it has the property of being stochastic or “memoryless”. The Monte Carlo class of computer algorithms is then used to sample the probability distributions and identify the most likely states.

HMMs were first introduced into biology in the late 1980s and were soon used to generate signatures of protein domains. For biological HMMs, every amino acid at each position in the multiple sequence alignment is given a score. Highly conserved residues are assigned high positive scores, weakly conserved residues have scores near zero and variable residues have high negative scores such that the sum of all probabilities is one. The power of the method may be enhanced through iteration of the search process. In a HMM search, the probability of a given sequence matching the HMM generated from a multiple sequence alignment is calculated and the matches ranked accordingly. In homology identification the usefulness of the Markov Chain Monte Carlo method is its application to evolutionary distant organisms where no assumptions need to be made about their relatedness. HMMs have the added benefit that the process usually favors functionally important residues since they are likely to be more conserved. This is not always the case though, particularly in divergent organisms or when proteins may have a common ancestral molecule but have evolved different functions.

A possible downfall of this method may arise when the majority of sequences in the multiple alignment used to generate a HMM are taken from closely related organisms. This overestimates the degree of evolutionary conservation of the residues common to the closely related organisms leading to a bias in the probability distributions and a decrease in the sensitivity of the model. We have found it useful to build HMMs from orthologs that are available from as many diverse organisms as possible (Durand et al. 2006), which increases the sensitivity of the model and this will be particularly important in the search for genes and proteins that are implicated in apoptosis in unicellular organisms. It is also recommended to increase the model’s specificity by incorporating experimental data. The homology search can be modified by weighting sequences in favor of functionally important amino acid residues as demonstrated from crystal structures and mutation data. These modifications will allow the researcher to identify homologs that may otherwise be missed. For a given gene or protein, the sensitivity and specificity need to be balanced according to available knowledge and the researcher’s objectives. A User’s Guide, the theory behind profile HMMs and the software for generating HMMs are freely available from the HMMER homepage.

### Interpretation of data

The accuracy of results is naturally dependant on the quality of the input data (see “Potential Pitfalls” later) and sequence homology data should always be interpreted with respect to the biology of the gene or protein being investigated. Due to the biological phenomena of parallel and convergent evolution, “similar function” is not necessarily conditional on “similar sequence” and nor does “similar sequence” confirm “similar function”. Parallel evolution occurs when two unrelated molecules evolve similar functions over similar time periods due to similar selective pressures; whereas in convergent evolution the time periods do not overlap (Zhang and Kumar, 1997). In both instances the molecules with overlapping functions evolve from different ancestral molecules and as a result may have, in part, similar sequences despite being unrelated. Such molecules are strictly non-homologous since they do not share an ancestral state, although they may be considered “functional homologs” if they perform similar functions in different organisms. It is also important to consider the corollary of this in which proteins with a common ancestor may have evolved different functions but their common ancestry is still reflected in their sequences. These molecules may erroneously be labeled as homologous due to the sequence similarity but are functionally divergent enough to be non-homologous.

By convention, expectation (E) values of ≤10^−4^ are considered statistically significant (an E value of 10^−4^ means that the probability of a match being identified by chance alone is 1 in 10000). This is not absolute however, and an inspection of the results is necessary before drawing conclusions.

### Databases

Nucleic acid and protein databases comprise an enormous repository of information and are easily accessible over the internet (Table 3). Most of these have cataloged data according to a specific system and are an essential resource when gathering information about the genes and proteins being investigated. A working knowledge of some of the most comprehensive databases is necessary to avoid duplicating information. For example, conserved domains, HMMs, multiple sequence alignments, molecular structures and other information for many of the genes and proteins involved in the initiation and execution of apoptosis in metazoans are likely to be available in various databases. This information may be downloaded and if necessary updated by the researcher and used as a starting point for the identification of homologs in protozoans.

### Identifying caspases in protozoans with homology methods

The identification of putative metacaspases in the protozoan *P. falciparum* demonstrated the potential of homology methods (Meslin et al. 2007; Wu et al. 2003). Two cysteine proteases were previously annotated in the *P. falciparum* genome database. Meslin et al. (2007) focused on gene PF13_0289 which harbored the catalytic dyad histidine and cysteine residues that are a signature of caspases. A multiple sequence alignment of various other putative caspases in plants and protozoans was performed with ClustalW (Thompson et al. 1994) and a highly conserved domain was identified that spanned ~144 amino acids. A HMM of this domain was used to search the *P. falciparum* genome with the HMMSEARCH program (Eddy, 1998) and the same PF13_0289 gene was identified with an E value of 2.5e-40 which confirmed the annotation as a cysteine protease. The rest of the molecule showed very little similarity to other caspases; however a region within the N-terminus attracted the authors’ attention. An inspection of the PF13_ 0289 amino acid sequence revealed several important hydrophobic residues typical of the CARD domain. A multiple sequence alignment of known CARDs identified a conserved 76 amino acid block and although the region in PF13_0289 demonstrated only weak homology with the conserved block, it was investigated further in the laboratory and phylogenetically. The steps taken in the identification and bioinformatic investigation of PF13_0289 are outlined in Figure 3.

The bioinformatic identification of metacas-pases in protozoans has been supported experimentally in *Plasmodium*, *Trypanosoma*, *Dictyostelium*, *Leishmania*, *and* yeasts (reviewed in Debrabant et al. 2003), which provided laboratory evidence for the bioinformatic data. Caspase-specific substrates were used to demonstrate a link between caspase activity and apoptosis, where the apoptosis phenotype was demonstrated by DNA fragmentation, mitochondrial membrane depolarization and phosphatidylserine exposure on the cell surface. The corollary of this was also demonstrated when caspase-specific inhibitors diminished or prevented apoptosis following induction of PCD, providing further evidence for the central role of caspase activity in PCD in these organisms.

## Phylogenetic Methods

A number of phylogenetic methods may be used to identify homologs and provide further evidence for their relationships. Various mathematical models that differ in their assumptions, parameters and types of input data, have been used to generate computer algorithms to determine the relationships between genes, proteins and organisms. The commonly used methods for inferring phylogenetic relationships are distance-matrix, maximum parsimony, maximum likelihood, and Bayesian inference methods. The distance-matrix methods rely on the “genetic distances” between the sequences being investigated where the distance is calculated from the proportion of mismatches in a multiple sequence alignment. The commonly used neighbor-joining algorithm is an example of this method. The maximum parsimony method constructs a phylogenetic tree that requires the least number of evolutionary events to explain the observed sequence data. The maximum likelihood method uses a mathematical model to infer probability distributions for possible phylogenetic trees where the probabilities are determined from a substitution matrix that takes into account differences in the rates of particular substitutions. Finally, Bayesian inference identifies the most probable tree given the data and a pre-determined evolutionary model. For comprehensive and authoritative reviews of the phylogenetic methods mentioned here, see Felsenstein (2003) and Holder and Lewis (2003).

Phylogenetic methods may be used to compliment homology methods. Evolutionary theory predicts an inverse relationship between evolutionary distance and sequence similarity (ED α 1/S or ED = μ/S where ED is evolutionary distance, μ is the mutation rate and S is percentage similarity) and it follows therefore that the sensitivity of a homology search of a particular genome increases if the sequences of closely related organisms are used in the search. Phylogenetic methods may be used to identify the most closely related organisms to the organisms being probed. Sequences from closely related organisms can then be used in the subsequent homology search. Similarly, the results of a homology search may be validated by constructing a gene tree of the results of the best E values in different organisms and comparing that to a species tree for the same set of organisms. The two trees should theoretically be in agreement. A useful resource that lists the available phylogenetic software may be found at http://evolution.genetics.washington.edu/phylip/software.html.

### Orthology and paralogy

As in most organisms, the apoptosis machinery in protozoans is likely to comprise several similarly functioning enzymes and proteins, such as the caspase and kinase protein superfamilies. Phylogenetic analysis is useful to differentiate orthology and paralogy (Dufayard et al. 2005; Gabaldon, 2005). The essential difference is that true orthologs occur by speciation events while paralogs occur by gene duplication events. This distinction is important because orthologous sequences are far more reliable when predicting protein functions than paralogous sequences (Gabaldon, 2005). Before inferences may be made about the functions of components of the apoptosis machinery in protozoans, knowledge of the evolutionary history of the components in terms of speciation and duplication events is therefore important. Dufayard et al. (2005) have developed an ingenious method for differentiating orthologs and paralogs. The algorithm matches new gene data in a phylogenetic tree to a database that catalogs species trees and by reconciling the two, predicts which sequences are orthologs and paralogs. There is no database specifically designed for protozoa but the HOGENOM database is devoted to completely sequenced genomes which include several protozoa (Dufayard et al. 2005).

### Phylogenetics of the CARD domain in the putative *P. falciparum* metacaspase

Meslin et al. (2007) made use of the maximum parsimony method available as Phylo_win software (Galtier et al. 1996), to generate a phylogenetic tree of known CARD domains, including the possible CARD region in PF13_ 0289. This revealed that the possible PF13_0289 CARD domain clustered with other apoptosis caspases, as opposed to inflammatory caspases, providing supporting evidence that this is indeed an apoptosis-related CARD domain. The phylogenetic analysis also led the authors to conclude that PF13_0289 is a primordial ancestor (termed a “metacaspase”) of the caspase family in multicellular organisms (Wu et al. 2003; Meslin et al. 2007), which would explain the limited similarity identified with homology methods. The authors continued the bioinformatic characterization of this domain with structural methods (see below).

## Phylogenomics and Other Whole Genome Methods

Several methods have been developed to exploit the information available from whole genome sequences to infer functional relationships between proteins. These are: i) gene co-occurrence or whole genome phylogenetic profiling; ii) mirror tree; iii) conservation of gene neighborhood; and iv) gene fusion events (Shoemaker and Panchenko, 2007). The first two methods have greater sensitivity and are discussed here. In gene co-occurrence or phylogenetic profiling (Pellegrini et al. 1999) proteins are characterized based on their presence or absence in a list of whole genomes. Proteins with matching profiles imply that the molecules have co-evolved, which infers that they interact (Huynen and Bork, 1998). The general method was improved by using various metrics to quantify the similarities between profiles and at the same time take into account the underlying phylogeny to differentiate between truly co-evolving genes and those that are merely present in a phylogenetically-related genome cluster. These improvements were often impractical as they required significant computational resources. Recently an improved phylogenetic profiling method has been published and demonstrated to be more computationally efficient (Cokus et al. 2007). It quantifies the similarity between profiles by determining the probability of two profiles having a certain number of matches based upon the number of proteins in each genome and at the same time accounting for the underlying phylogeny by ordering genomes according to their similarity. The limitations of this method are that it can only be applied to complete genomes and cannot be used with proteins that are common to most organisms.

A second approach for identifying protein interactions is the “mirror tree method”. This method is based on the finding that the phylogenetic trees of interacting proteins demonstrate a greater degree of similarity than non-interacting proteins. Stated another way, the correlation between the distance matrices of pairs of proteins is a good predictor of functional relatedness (Pazos and Valencia, 2001). This approach is limited by the potential inaccuracies of the multiple sequence alignment used to build the phylogenetic trees.

The benefit of phylogenomic methods for studying PCD lies in the fact that the apoptosis machinery involves a cascade of protein interactions suggesting intimate co-evolutionary relationships. These methods are currently very useful as an adjunct to other approaches mentioned in this review. Once a protein that plays a key role in PCD has been identified, it may be possible to rapidly identify other components involved in the apoptosis pathways. This approach will also become more useful as more complete genomes become available. For protozoans these methods may be useful for the apicoplast group as there are a number of complete genomes available including *Plasmodium*, *Theileria* and *Babesia* species.

Some of the PCD effectors and regulators such as the Ap-ATPases have an unusual evolutionary history in that the gene and species phylogenetic trees do not match, which suggests that some components of the apoptosis machinery were acquired by horizontal gene transfer (HGT) (Aravind et al. 1999). In the case of Ap-ATPases, it is believed that the HGT arose from gram positive bacteria. This feature may be used to identify other apoptosis genes that were acquired similarly. Foreign genetic elements often have a different nucleotide usage profile to the rest of the genome in which they find themselves. Reva and Tümmler (2005) have used oligonucleotide usage patterns to differentiate foreign sequences in prokaryotes. Whether this can be extended to eukaryote genomes is currently being investigated (Reva, personal communication) and may assist in identification of components of the apoptosis machinery that have been derived via HGT.

### The use of phylogenomics to identify protozoan proteins involved in apoptosis

The value of these methods lies in the potential identification of proteins that have co-evolved with a candidate apoptosis-related protein. Phylogenomic methods were unnecessary for the initial identification of the ancestral metacaspase PF13_0289 (Meslin et al. 2007) but by using the methods described above, the investigators may have identified molecules that interact with it. The phylogenetic profiling developed by Pellegrini et al. (1999) has been implemented in the Plasmodium Genome Resource Database (www.plasmodb.org) but no proteins with profiles significantly similar to PF13_0289 have yet been identified. The most likely reason for this is that orthologs of PF13_0289 have not been found in the complete genomes that are available for other protozoans. Once proteins that are predicted to interact with PF13_0289 are identified, they may be investigated more closely for the presence of apoptosis-related signature domains. This will potentially identify other proteins involved in the apoptosis pathway or even identify a novel pathway in protozoans.

## Sequence Evolution: Rates and Constraints

The rates of nucleotide sequence evolution enable a further method for the identification of homologs and differentiating orthology from paralogy. The ratio of non-synonymous (K_a_) to synonymous (K_s_) nucleotide substitution rates is predictive of stabilizing or diversifying selection (Yang, 2002) and should theoretically be the same for orthologs but may differ for paralogs. The PAML (Phylogenetic Analysis by Maximum Likelihood) system provides methods to identify positive or stabilizing selection at particular codon sites or across lineages (Yang, 1997; Yang and Swanson, 2002) and has been used extensively for sequences that are known to be orthologous (Yang, 2002) or for sequences within a species, viral subtype or strain (e.g. Soeighe et al. 2007). The authors are not aware of any examples in the literature where PAML has been applied to situations where orthology was not confirmed. However, we predict that this may be a useful tool for generating supporting evidence for orthology when evolutionary distances are so great that homology methods are not sufficient on their own. Proteins that are under the same evolutionary constraints are likely to demonstrate the same pattern of selection across individual codon sites even if the primary sequences have evolved to a point where the sensitivity of the homology methods is insufficient to predict orthology. A comparison between the patterns of sequence evolution across codon sites of the known orthologs and the putative orthologs will predict potential similarities in structure and function. This process should also reveal information about sites of particular functional importance. The same principle can be applied to variations in mutation rates across lineages where the pattern of sequence evolution will provide information about the selective forces acting on the sequence. We are currently investigating the use of patterns of sequence evolution as a method for determining homology.

### Sequence evolution in metacaspases

Due to the evolutionary conservation of caspases, it is reasonable to expect that their patterns of non-synonymous and synonymous nucleotide substitutions would be similar. Functionally important codons usually demonstrate a pattern of stabilizing selection (Massingham and Goldman, 2005). The investigation of the candidate metacaspase PF13_ 0289 identified by Meslin et al. (2007), may be refined by examining the nucleotide substitution pattern of this gene. This level of bioinformatic characterization is helpful for inferring functional similarities between proteins and will strengthen the evidence for the involvement of the candidate gene in initiation or execution of apoptosis in protozoans.

## Structural Methods

Following the identification of putative homologs, determination of the molecular structural characteristics using computational methods will provide more direct evidence for functional homology. One approach is to model the putative homologs using the resolved crystallographic or NMR 3D protein structures of the most closely related organisms as templates and determine the predicted thermodynamic stability and probability of the putative structures using computer algorithms. The worldwide protein data bank (Berman et al. 2003) is a global archive of macromolecular structural data which may be used to predict structures of related molecules with available software like SWISS-MODEL (Schwede et al. 2003). Predicted models with high probabilities and thermodynamically stable structures are likely to have similar functions as the template. A number of the apoptosis proteins have distinct structural features, which would be useful for inferring function in homologs. For example, the DD, DED and CARD domains all have similar α-helical folds and it is hypothesized they evolved from a common ancestor. These domains are present in many apoptosis proteins including initiators and executors and help glue the apoptosis proteins together (Hofmann et al. 1997). Similarly, all caspases have a distinct structural fold that is essential for their catalytic function and may be used as a structural signature of their function (Thornberry and Lazebnik, 1999).

An alternative approach using structural methods is to determine whether there are any structures that have a primary sequence similar to putative homologs. One way to do this is to make use of the large number of available predicted structures. The newly identified putative homolog is used to query a structural database to identify proteins with a similar primary sequence. The inference is that the query sequence will exhibit a similar structure to the matched proteins. This method can be performed with SCOP, Structural Classification of Proteins Database developed by the Cambridge Centre for Protein Engineering (Murzin et al. 1995). The unit of structure identification used by SCOP is a discrete domain instead of a whole protein which increases the accuracy and sensitivity of the investigation. SCOP also demonstrates that the combination and arrangement of individual domains within a protein provide further evidence for orthology. Two proteins with domains arranged in the same order are more likely to be orthologous even if they have significantly divergent sequences, than two proteins with more similar primary sequences but where domains are arranged in a different order. In the latter case, the two proteins are more likely to be paralogous.

The protein-protein interactions of resolved and predicted structures may also be studied computationally to analyze the components of a cascade or signaling pathway such as those that occur in apoptosis (for a review of the methods and available software see Piehler, 2005).

### Structure of the CARD domain of the putative *P. falciparum* metacaspase (PF13_0289)

The CARD domain structure of PF13_0289 was modeled using a known CARD as a template (Meslin et al. 2007). The most likely model consisted of 6 anti-parallel α-helices surrounding a hydrophobic core, the typical topology of CARD. This provided evidence that PF13_0289 is a caspase enzyme with a CARD domain. The combination of homology methods, phylogenetics and structural methods provided a comprehensive computational characterization of PF13_0289 and extensive evidence that it is an ancestral metacaspase molecule with cysteine protease and CARD domains.

## Potential Pitfalls

A thorough knowledge of the biology of the genes and proteins being investigated is imperative before a bioinformatic approach is undertaken. This will diminish the likelihood of erroneous results by verifying input data and detecting inaccuracies due to incorrect assumptions or limitations of the computational methods used.

### Input data

The first and most important potential pitfall is the quality of the primary input data. For PSI-BLAST, hidden Markov models and other homology methods, input data must be trimmed to exclude untranslated or contaminating sequence. A good resource for reference sequences is RefSeq (Pruitt et al. 2000) which was developed to overcome the confusion that results when several entries exist in databases for a given gene or protein. RefSeq entries are classified as reviewed, provisional and predicted with those designated “reviewed” indicating an extensive manual review process to exclude contaminating or vector sequences and to ensure that the gene or protein sequence is not truncated. For unicellular eukaryotes, much of the data has not been reviewed and the researcher needs to critically examine sequences to verify the quality. It is essential to know whether the input data contains whole genes, transcribed sequences, discrete domains or complete protein sequences. For gene transcripts, it is important to verify that the complete 5′ end is included by identifying a start codon. The sequence should be trimmed to exclude untranslated regions before and after the start and stop codons prior to using it as input data. Errors in bioinformatics tend to accumulate and amplify as new work makes use of published data that may contain small inaccuracies. As far as possible, preference should be given to experimentally verified information to avoid error magnification.

### Multiple sequence alignments

The MSA is possibly the most critical step as it forms the basis for generating Markov models, phylogenetic and phylogenomic analyses, and investigating sequence evolution. For simple cases where sequences do not contain indels and there is only one well-conserved domain, different algorithms are likely to give the same result. However, for problematic sequences careful consideration must be given to the algorithm being used. The authors of this review have found the MAFFT method (Katoh et al. 2002) particularly useful as one can select a variety of algorithms depending on whether the sequences contain one or more domains, several conserved regions embedded in unalignable regions, flanking sequences, or indels.

### Output

The significance of homology matches should always be interpreted in conjunction with biological relevance. The E value in BLAST searches is generally a good predictor of similarity but does not take into account the biological relevance of specific residues which may be essential or unimportant for function. It should be remembered that the output is a measure of statistical similarity which frequently, but not automatically, correlates with functional similarity. In addition, gene expression does not necessarily translate into functional similarity: a correlation coefficient of 0.48 was found between the expression of genes and their respective proteins (Anderson and Seilhammer, 1997). Finally, the computational process itself contains inaccuracies and should be investigated if results cannot be explained (Bork, 2007).

### Conventional methods and unconventional genomes

Unconventional genomes present further challenges for an investigation into unicellular eukaryotes. The human malaria parasite *P. falciparum* contains the most unusual genome sequenced to date (Gardner et al. 2002), e.g. it is the most AT-rich genome identified and sequences contain numerous low complexity regions. This makes the application of conventional bioinformatic methods more difficult, which was reflected by the fact that less than half of the genes had predicted functions when the complete genome was released. In investigations of the *P. falciparum* genome, the authors of this review have used two approaches to improve the accuracy of the computational methods (Durand et al. 2006). Firstly, a detailed analysis of the biologically important residues and a weighting in favor of these sites when generating hidden Markov models identified potential homologs that would otherwise be missed based purely on E-values. Secondly, BLAST analysis gives the option of a low complexity filter which masks low complexity regions from sequences during a search. This may be applied to nucleotide sequences or amino acid sequences. In *P. falciparum* this is important as there are long stretches that are AT-rich in nucleotide sequences and homopolymeric runs of lysine, arginine or glutamine in proteins.

In unusual and distantly related genomes, it is even more important to use conserved regions or domains as queries rather than complete sequences in order to minimize the effects of sequence divergence and recombination.

## Verification of Bioinformatic Data

In its most basic form, bioinformatics uses gene and protein sequences to make predictions about similar genes and proteins. Following the sequencing of complete genomes and advances in other areas of biology such as structural biology, molecular evolution and systems biology, computational methods were developed to analyze and generate hypotheses concerning the information present in the available data. However, the analysis that is generated from computational methods is largely theoretical or predictive in nature. Due to the inevitable inaccuracies in the methods employed, variations in biological systems and phenomena such as parallel and convergent evolution, bioinformatic data must be interpreted with caution. Laboratory investigation is usually required to verify that the genes and proteins identified bioinformatically, are indeed expressed in the relevant cells and that they play a role in PCD.

## Concluding Remarks

PCD in protozoans has largely been limited to studying the inducers of apoptosis and the resultant phenotype, while very little is known about the initiators and executors that comprise the apoptotic machinery. The bioinformatic approach offers an array of extremely powerful methods for this purpose. This review illustrates how they may be combined for identifying the evolutionarily divergent apoptosis machinery in protozoans, using the computational characterization of a metacaspase gene in *P. falciparum* as an example.

## Figures and Tables

**Table 1:** Key concepts.

**Apoptosis**	The most typical phenotype of PCD, characterized by specific morphological and biochemical features
**Diversifying selection**	An evolutionary process by which genetic diversity increases over time
**Expectation (E) value**	The degree of similarity one expects to find between two genes or proteins by chance alone
**Hidden Markov model**	A probability model in which a system is modeled using the Markov process to identify hidden parameters using observable data
**Markov property**	A system whereby any given state is conditionally independent of any prior states
**Ortholog**	A homologous gene or protein that has arisen as a result of speciation
**Paralog**	A homologous gene or protein that has arisen as a result of gene duplication
**Programmed cell death (PCD)**	The inherent capacity of a cell to implement an active and regulated mechanism of self-destruction
**Stabilizing selection**	An evolutionary process by which genetic diversity decreases over time

**Table 2:** The cellular machinery involved in apoptosis.

Second and third generic stages of PCD	Molecular components of PCD	Examples	Domains/Conserved regions
**Initiation**	Receptors	Toll-like receptors, TNF-like receptors, FAS, IL-1	DD, TIR, FNIII, Ig fold
	Transcription factors	Cell cycle control proteins, MYC, tumor suppressors	DBD, Ig fold
	Other intracellular and	Receptor associated signaling	MATH, CART, DD, Ig
	nuclear proteins	molecules, cyclin, CDKs, NF-κB, kinases, TRAF	fold
**Execution**	Effectors	Caspases and other cysteine proteases, RIP kinase, BAX, BCL-2	Casp, DD, CARD, DED, kinase domain
	Regulators	AIPs, Ap-ATPases, NTPases, serine/threonine kinases	conserved motifs in Ap-ATPases, Zn finger

**Notes:** The molecular components with examples of the second (initiation) and third (execution) stages of PCD are listed. The domains and conserved regions that may be useful for the bioinformatic identification of homologs of the apoptosis machinery in unicellular eukaryotes are indicated.

**Abbreviations:** AIPs: apoptosis inhibitory proteins; Ap-ATPases: apoptotic ATPase; BCL-2: B cell leukemia/lymphoma 2 gene; CARD: caspase activation and recruitment domain; CART: cysteine-rich motif associated with RING and TRAF; Casp: caspase catalytic domain; CDK: cyclin dependant kinase; DBD: DNA-binding domain; DD: death domain; DED: death-effector domain; FNIII: fibronectin domain; Ig: immunoglobulin; IL-1: interleukin 1; MATH: meprin and TRAF homology domain; MYC: myelocytomatosis viral oncogene homolog; NK-κB: nuclear factor-κB; TIR: toll-interleukin-receptor domain; TNF: tumor necrosis factor; TRAF: TNF receptor associated factor.

**Table 3:** Computational methods for identifying and investigating homologous genes and proteins.

Method	Description	Examples
Homology search	Profile-based algorithm to search for homologous sequences	Gapped BLAST, PSI-BLAST
Multiple sequence alignment (MSA)	Alignment of multiple sequences to maximize the homology between them	CLUSTAL, Muscle, PileUp, T-Coffee, MAFFT
Hidden Markov model	Probability distribution across all residues in a MSA to generate a pattern of conservation or signature profile	Markov Chain Monte Carlo
Phylogenetics	Determination of the evolutionary relationships between taxa	Distance-matrix, maximum parsimony, maximum likelihood, Bayesian inference
Phylogenomics	Whole genome comparisons for identifying protein-protein interactions based on various criteria	Phylogenetic profiling, mirror tree, gene neighborhood, gene fusion
Sequence evolution	Identification of patterns of positive and stabilizing selection across phylogenetic lineages and codon sites	Phylogenetic analysis by maximum likelihood
Structure analysis	3D structure prediction	SWISS-MODEL

**Table 4:** Useful websites for bioinformatics researchers.

European bioinformatics institute (www.ebi.ac.uk)	A comprehensive resource of all bioinformatic data, includes a variety of bioinformatic tools such as programs for MSAs (CLUSTAL, MAFFT etc) and structural analysis.
HMM homepage (http://hmmer.janelia.org)	Downloads of available HMM software, includes a user’s guide and theoretical information.
HOGEMON database (http://pbil.univ-lyon1.fr/databases/hogenom.html)	Database of homologous sequences from fully sequenced genomes.
InterPro (www.ebi.ac.uk/interpro)	Integrates information from numerous other protein databases.
National center for biotechnology information (http://www.ncbi.nlm.nih.gov/)	National resource of molecular biology information, includes bioinformatic tools, PubMed.
Plasmodium database (www.PlasmoDB.org)	Database of *Plasmodium* bioinformatic data.
Pedant phylogenetic web profiler (http://pedant.gsf.de)	Web-based service to perform phylogenetic profiling of proteins against genomes.
PLATCOM (http://platcom.informatics.indiana.edu/platcom)	Integrated system for comparison of multiple genomes.
Protein analysis through evolutionary relationships (www.pantherdb.org)	Classification of genes by function, phylogenetic trees, HMMs and multiple sequences available.
Phylogeny resource (http://evolution.genetics.washington.edu/phylip/software.html)	Comprehensive list of available phylogeny software, software downloads available.
ProDom (http://prodom.prabi.fr/prodom/current/html/)	Comprehensive set of protein domain families.
Pfam (www.sanger.ac.uk/Software/Pfam/)	Collection of multiple sequence alignments and HMMs covering protein families.
ProSite (http://au.expasy.org/prosite/)	Database of protein families, domains and functional sites.
Protein data bank (www.wwpdb.org)	Archive of macromolecular structural data.
Reference sequence collection (http://www.ncbi.nlm.nih.gov/RefSeq/)	Collection of non-redundant sequences that have been expertly reviewed.
STRING (http://string.embl.de/)	Database of known and predicted protein-protein interactions.
Structural classification of proteins database (http://scop.mrc-lmb.cam.ac.uk/scop-1.61/index.html)	Classification of proteins based on structural similarities.
Universal protein resource (http://www.expasy.uniprot.org/)	Catalog of proteins, integrates information from Swiss-Prot (curated expert knowledge base), TrEMBL (non-curated computer annotated supplement to Swiss-Prot) and PIR (protein sequence database), links to protein analysis tools such as SWISS-MODEL.
